# What’s Next for Microalgae Oil? A Scientific Mapping for Saturated Fatty Acids

**DOI:** 10.3390/foods14193451

**Published:** 2025-10-09

**Authors:** Michelle Amario, Daniel Kurpan, Wendel Batista da Silveira, Anita Ferreira do Valle

**Affiliations:** 1 Laboratório de Bioquímica e Biotecnologia de Algas, Departamento de Bioquímica, Instituto de Química, Centro de Tecnologia, Universidade Federal do Rio de Janeiro, Ilha do Fundão, Rio de Janeiro 21941-909, RJ, Brazil; mia.ufrj@gmail.com; 2 Food Microbial Systems, Agroscope, Schwarzenburgstrasse 161, 3003 Bern, Switzerland; daniel.kurpan@agroscope.admin.ch; 3 Laboratório de Fisiologia de Microrganismos, Departamento de Microbiologia, Instituto de Biotecnologia Aplicada à Agropecuária, Universidade Federal de Viçosa, Viçosa 36570-900, MG, Brazil; wendel.silveira@ufv.br

**Keywords:** microalgae, food, lipids, saturated fatty acids, monounsaturated fatty acids, polyunsaturated fatty acids, extraction methods

## Abstract

Lipids obtained from microalgae have recently received significant attention from the energy and food industries. Microalgae are promising alternatives and are more sustainable sources of lipids for the food industry, which faces a growing demand for food and increased environmental awareness among consumers. This study provides a bibliometric review of research articles published between 2019 and 2024 with the aim of understanding the future trends and tendencies of the applications of microalgal lipids in the food industry. A thorough assessment of 255 articles retrieved from the Scopus database showed an apparent decrease in the number of publications per year within the analyzed timeframe. The predominant focus has been basic research conducted on a lab-scale using chlorophytes (green algae) to optimize lipid production by modulating physicochemical cultivation parameters (i.e., nutrient availability, temperature, light, and pH). Lipids were mainly extracted using the Bligh and Dyer or Folch methods, quantified gravimetrically, and characterized using gas chromatography coupled to mass spectrometry. Publications referring to polyunsaturated fatty acids, such as omega-3 and omega-6, were the most abundant. The results emphasized the significance of microalgae as a promising biotechnological platform for the production of lipids within the food industry.

## 1. Introduction

The global food industry is vast and highly diverse, encompassing activities that range from agriculture to processing, distribution, and retail. In recent years, however, in-creasing attention has been drawn to the environmental impacts associated with this production chain [1,2]. Among the most pressing issues are deforestation and biodiversity loss, largely driven by the expansion of agricultural frontiers for crop cultivation and live-stock production [3,4].

Food systems are also major contributors to greenhouse gas (GHG) emissions [5], with livestock being a primary source of methane (CH_4_) [6], intensive fertilizer use releasing nitrous oxide (N_2_O) [7], and carbon dioxide (CO_2_) emissions arising from food production and transportation [8]. In addition, agriculture accounts for nearly 70% of all global freshwater withdrawals, placing immense pressure on rivers, lakes, and aquifers [9]. Excessive use of pesticides and fertilizers further contributes to soil degradation and environmental pollution [10], exacerbating the ecological footprint of food production [11].

There is a growing consensus that food production will need to increase substantially in the coming decades to meet the demands of a rising global population, but doing so requires minimizing environmental impacts to ensure long-term sustainability. In this context, sustainable agriculture approaches such as agroforestry have been introduced, emphasizing not only the need to boost food production but also to reduce pollution, avoid uniform fertilizer and pesticide use, encourage site-specific practices, enhance nutrient-use efficiency, decrease nitrogen application, and safeguard agriculture reliant on natural resources [2,12]. This challenge calls for a process known as ‘sustainable intensification’ [13], which encompasses the optimization of resource use, the integration of innovative technologies, and advances in genetic improvement.

At the same time, global consumption patterns are shifting, as consumer choices are being increasingly driven by ethical concerns about animal welfare and the environmental impacts of meat consumption [14,15,16]. Growing awareness of animal abuse in industrial farming, alongside broader ethical debates, has led many individuals to adopt flexitarian or fully vegan diets [17,18]. These dietary shifts reflect a conscious effort to reduce reliance on animal-based products while aligning food consumption with personal values of compassion, sustainability, and social responsibility [19]. Consequently, the demand for plant-based food products is increasing [20].

Combined, these factors are driving a significant transformation in the food industry, driven by shifting consumption patterns, government incentives and regulations, and technological innovations. The food industry’s response to environmental and social concerns has been driven by innovation at various stages. For example, large biotechnology companies have invested in new technologies to produce food ingredients in the plant-based meat product sector. In this scenario, microalgae have gained prominence as potential sources of compounds to be used by this industry [21,22]. In contrast to traditional crops, microalgal cultivation requires less water and can take place in controlled environments, such as photobioreactors, located on non-arable land or underutilized urban areas. These closed systems allow large-scale cultivation without the need for large tracts of land, reducing pressure on natural ecosystems and avoiding the conversion of natural habitats to agricultural land. Moreover, microalgae can produce biomass with high added value because it contains pigments, antioxidants, fatty acids, and vitamins [23,24]. These advantages have attracted the attention of several commercial sectors such as the food industry, as evidenced by the increasing number of studies on the use of microalgae as a functional food in recent years [25]. Microalgal derivatives can be marketed as dietary supplements (powder or oil), ingredients, or additives [26]. Advances in the conversion of microalgal biomass have led to new food products and ingredients for beverages, bread, and pasta [27,28]. In addition, microalgae are emerging as a promising source of bioavailable proteins [29].

Of particular interest in this work, microalgae produce a broad range of lipids that have numerous applications in the food industry. These lipids can be extracted and used as functional ingredients in a variety of food products, including processed foods, dietary supplements, functional foods, and fortified formulations [30,31,32]. Major lipids include polyunsaturated fatty acids (PUFAs), such as omega-3 and omega-6 [33]. Among the long chain PUFAs, eicosapentaenoic acid (EPA) and docosahexaenoic acid (DHA) are known for their health benefits, particularly in the prevention of cardiovascular disease and brain development [34]. In addition, microalgae produce triacylglycerols (TAGs), which serve as a source of energy in dietary ingredients [35]. Microalgal TAGs contain fatty acids such as palmitic acid (C16:0), used in the production of modified oils and fats; oleic acid (C18:1), used as functional oils and emulsifiers; linoleic acid (C18:2), used in functional foods and dietary supplements; and arachidonic acid (C20:4), also used in functional foods and product formulations intended for children given its role in cell development and health [33]. Microalgae also produce phospholipids, which have emulsifying properties and are widely used in the formulation of processed food [36]. Carotenoid pigments such as astaxanthin and lutein are valued for their antioxidant properties and ability to act as natural colorants [37]. This group is commonly used in foods and beverages to improve the nutritional and aesthetic quality of the final product [23]. In addition to the potential of polyunsaturated fatty acids, our study seeks the state-of-the-art knowledge of saturated fatty acids from microalgae that can replace saturated fat from animals and plant-based foods.

Given the current demands that environmental and social changes imposed upon the food industry, this work aims to map the use of microalgae lipid in this field, under-standing the structure, trends, and gaps in academic literature. This work presents the results of a bibliometric review on the production of lipids by microalgae and their applications in the food industry. It describes (i) the articles published in peer-reviewed journals, (ii) the organisms studied, and (iii) the main methodologies that have been used in the past six years (2019–2024). The main current trends in the field are discussed and, finally, conclusions are drawn to guide future research.

## 2. Materials and Methods

### 2.1. Systematic Search and Selection Criteria for Scientific Papers

A systematic search of research articles in the Scopus database (www.scopus.com) was conducted to describe the state of the art in the scientific production and use of oils obtained from microalgae, covering the period from 1 January 2019, to 31 December 2024. Scopus was chosen for this bibliometric review because it balances broad multidisciplinary coverage with consistent metadata quality, robust citation analysis tools, and flexible export options, which ensured reliable data extraction and facilitates accurate analyses.

Titles, abstracts, and keywords of peer-reviewed articles were searched using eight selected keywords combined in different ways (Table 1) to retrieve publications relevant to this study, leading to performing 20 searches on the database. All bibliometric research was conducted in Scopus using the Boolean operator ‘AND’ to combine keywords. Each query applied the TITLE-ABS-KEY field restriction, ensuring that retrieved documents contained the specified terms in the title, abstract, or keywords. The same date range (January 2019 to December 2024) was consistently used across all searches to maintain comparability of results.

From all searches, a total of 17,081 articles were retrieved and subjected to a screening process to identify those aligned with the aims of this study. Since the keywords used in the search were intrinsically related to microalgae lipid production, the first filtering step involved the removal of duplicates retrieved across multiple searches. Subsequently, a second round of keywords was applied using two approaches to further reduce the dataset. The first approach sought to exclude articles focused on broader application areas of microalgae and their derivatives, such as bioenergy, wastewater treatment, and cosmetics. To identify and remove these, related keywords (e.g., biofuels, bioethanol, biogas, waste, treatment, cosmetics) were searched within the titles and abstracts. The second approach aimed to retain articles most relevant to the study objectives, achieved by searching for the keyword “food” in titles and abstracts and keeping those records in the dataset. For the remaining articles that did not fit either approach, a preliminary screening of titles and abstracts was performed to assess their suitability for inclusion. Subsequently, the dataset was refined through an in-depth review of titles and abstracts to select the final articles. It should be noted that *Aurantiochytrium* spp. were excluded from this research, even though their oil is commercially referred to as “microalgal oil”. These heterotrophic protists were not considered microalgae in this study.

### 2.2. Data Collection for Mapping Microalgae Lipid Applications

From the final dataset, a detailed analysis was conducted to extract information on the microalgae species, methods, applications, and target lipid molecules investigated over the past six years. Understanding the species used was essential for identifying those with the greatest potential for food industry applications, while examining the methods highlighted current technological approaches and experimental designs. Analysis of tar-get lipid molecules revealed which bioactive compounds are being prioritized, and assessed applications provided insight into how these species and compounds are being translated into products related to the food industry.

Also, the bibliographic analysis was performed using Scopus tools to build the current scenario of the production of microalgae oil, considering the publication frequency, origin and subject area to which the articles were associated with. To access information regarding the country of origin, Scopus considers the attributed country of the affiliation institution of all authors for each publication [38], leading, in some cases, to the attribution of more than one country per publication. This evaluation allows not only inferences about country investments, leaders in a determined field and emerging players, but also reveals international cooperations as a reflection of policy priorities.

To identify the main field that are being pursued in the context of the production of microalgae lipids, and possible gaps of knowledge, an analysis of subject area information available through Scopus’ tools was conducted. In this case, Scopus assigns subject areas through the All Science Journal Classification (ASJC) system, where each journal receives one or more subject codes that are inherited by its articles, which standardize the output categories, allowing comparisons across disciplines and revealing each articles’ interdisciplinarity [39].

### 2.3. Data Organization and Analysis

All data collected was organized in spreadsheet files for analysis and are available for download (see the Supplementary Materials). Considering that in the majority of metrics analyzed, individual articles could be associated with multiple characteristics, the contribution was calculated based on the frequency of occurrence of each characteristic.

## 3. Results and Discussion

### 3.1. Description of Documents

**Figure 1:**
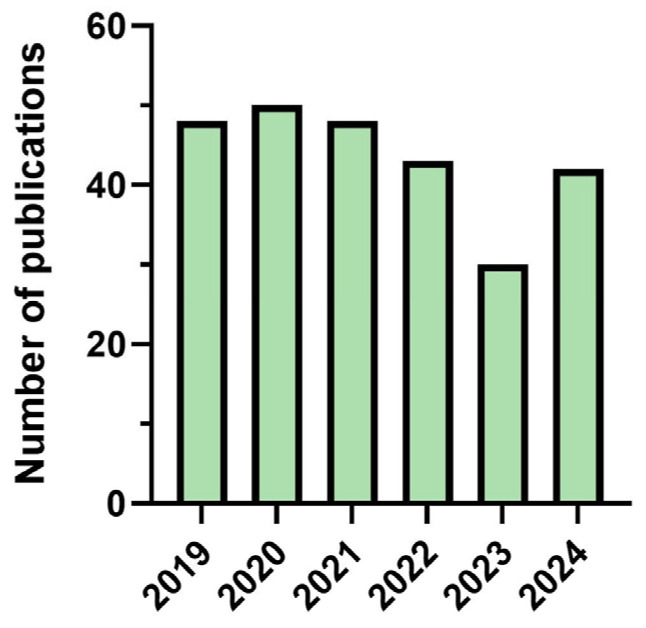
The annual number of scientific publications regarding lipid production from microalgae for the food sector.

The average number of publications per year was approximately 40 from 2019 to 2022. In 2023, there was a decrease in publications to 29, returning to the average of 40 in 2024 (Figure 1). The noticeable decline in the number of publications related to microalgal lipids in 2023, when compared to the period from 2019 to 2022, may be partially explained by broader trends in global research activity during and after the COVID-19 pandemic. Several studies have shown that the pandemic significantly influenced scientific publishing patterns. For instance, there was a redirection of research efforts and funding toward COVID-19-related topics, which led to an estimated 18% decrease in non-COVID-19 research output in leading medical journals [40].

The publications came from a total of 58 nations (Supplementary Materials), although they were mainly concentrated from a few countries (Table 2). China was the leading contributor with 13.1% of the publications, followed by Spain with 5.6%, and Brazil, Italy, and Portugal each with 5.3%. A total of 152 institutions, including universities, research centers, foundations, and government agencies, collaborated globally and actively participated in the prospecting study topic (Supplementary Materials).

When the 255 publications were classified based on the subject areas, some publications were related to more than one area, resulting in 459 occurrences distributed over 21 areas (Table 3). Among the subject areas, agricultural and life sciences stood out, accounting for almost 25% of the publications. This suggests that most of the research on microalgal lipids has focused on the biological characteristics of the organisms and on the optimization of cultivation conditions for the production of biomass and target compounds. In addition, the use of microalgae as a sustainable alternative for agricultural processes has received great attention recently [41]. Studies focusing on biochemical, genetic, and molecular approaches accounted for 13.5% of the publications. This finding reflects the growing interest in understanding the biochemical processes involved in the production and improvement of the target product. At the same time, advances in molecular and genetic techniques in recent decades have provided new tools for innovating and improving organisms, whether by modifying gene expression through epigenetics or by editing the genomes of microalgae strains in culture. The commercial use of such methods is still limited due to their high costs and a weak regulatory framework [42]. Chemical engineering and environmental sciences were the subject of 11.5% and 8.7% of the publications, respectively. These areas reflect the interest in using microalgae for technological purposes and as bioremediation tools, such as wastewater treatment. Recent studies have shown that the use of microalgae for effluent treatment, in addition to minimizing industrial damage to the environment, can increase the lipid production of the organisms due to the abundance of nutrients present in the effluent, thus reducing the costs related to maintenance and cultivation [43]. It is also worth highlighting the area related to biofuels and energy (8.1% of the publications): It has been one of the main drivers and motivators for the evaluation of microalgae [44]. Lastly, the area related to health, medicine, and pharmaceuticals, although still at an early stage of development, is contributing to the advancement of microalgal applications, in particular by evaluating their potential for producing bioactive compounds that can act as antiviral, antibacterial, and antioxidant agents [45,46].

The analyzed publications included a wide range of applications of microalgal lipids (Table 4). Almost 30% of the publications were basic research. In general, those publications have aimed to test different cultivation conditions to optimize and maximize biomass production or to stimulate the production of a specific compound. These studies are carried out by manipulating the main abiotic factors that influence the growth of microalgae—for example, light intensity, temperature, and the concentration of certain nutrients such as nitrogen and phosphorus [47,48]. Changes in the consumption patterns of societies interested in adopting a healthier and more sustainable lifestyle [49] are reflected in the classifications related to food, nutraceuticals, and pharmaceuticals, contributing 20.7%, 13.4%, and 5.1% of the publications, respectively.

The objectives of the publications were also analyzed (Table 5). Production and product improvement was the most addressed topic (20.4% of the publications), suggesting that a significant part of the research has focused on optimizing production processes and increasing the production of microalgal lipids. In addition, 20.0% of the publications focused on the characterization of lipid profiles. This suggests that it is not only the quantity of lipids produced that is of interest to the research community, but also an understanding of which complex mixture of compounds can be obtained. Other topics covered by the publications include production optimization of target compounds (15.1% of the publications) and the production of microalgal biomass (9.2% of the publications), indicating a search for greater productivity of specific molecules rather than the whole biomass. Several lipid extraction methods are currently available and widely used in research [50], and 7.2% of the publications examined which methods are optimal. The selection of the right extraction method depends on the desired target compound and its applications. In addition, 6.8% of the publications addressed the benefits of microalgae, including the benefits of these biomolecules for health or other applications. Finally, only 5.4% of the publications covered molecular mechanisms, indicating that the research on microalgal lipids has been more focused on applied research rather than on enhancing knowledge related to their metabolism.

**Figure 2:**
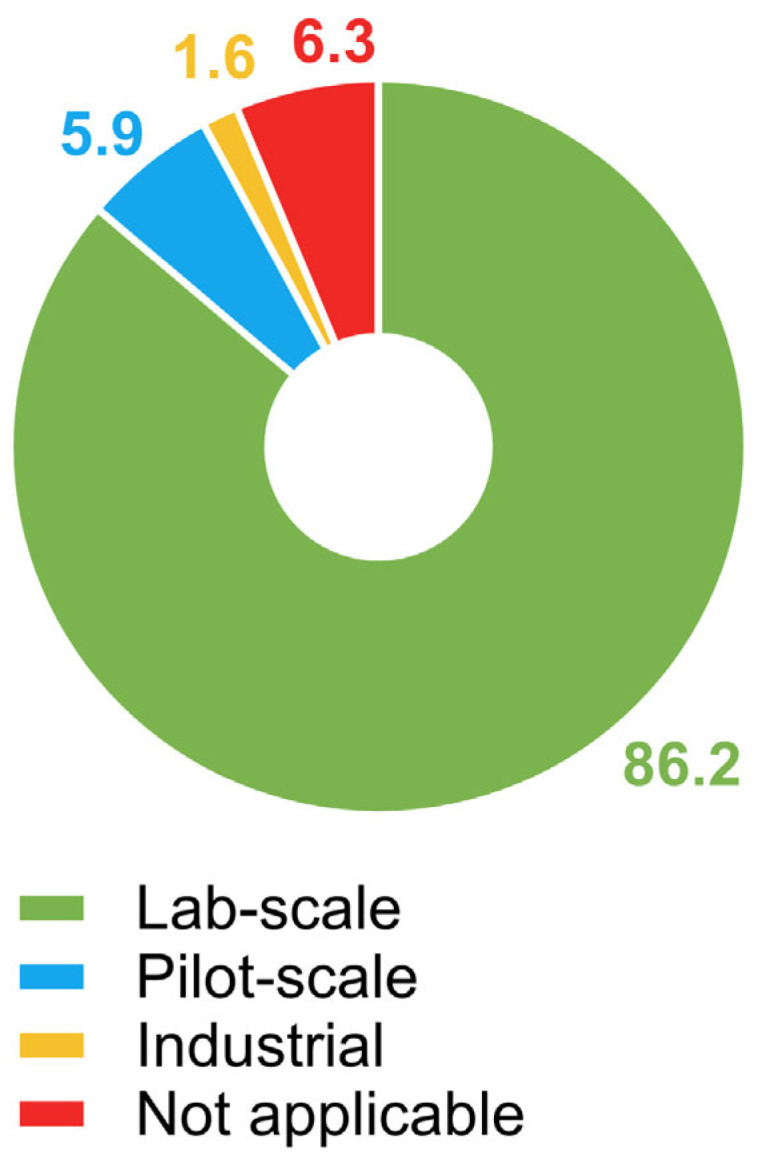
The distribution of publications based on the scale at which the study was carried out. “Not applicable” represents theoretical publications.

The scale at which the publications were developed was assessed because it represents an objective indicator of the technology readiness level (TRL) of the process and its feasibility for commercial application. Laboratory-scale studies are conducted in controlled environments where small volumes are used to test hypotheses, to validate methods, and to optimize process conditions. They are essential for the development of new technologies due to its relatively low cost and the possibility of rigorous control of experimental variables. The vast majority of the publications, almost 90%, conducted lab-scale research (Figure 2), possibly testing innovations experimentally before scaling up. At the pilot scale, processes are scaled up to a pre-industrial level to test their technical and economic viability under conditions that simulate large-scale production. Only 5.9% of the publications examined large-scale production, which may indicate an initial transitional stage that is crucial for adapting the process before its commercial application. The industrial scale involves large-scale production with a focus on efficiency and economy, with a market-directed view. This level requires complex infrastructure, great investment, and strict quality control. Only a small proportion of the publications (1.6%) involved real-scale, well-established technologies. Finally, 6.3% of the publications were not classifiable based on scale because they approached the subject in a theoretical way.

### 3.2. Organisms Characteristics

**Figure 3:**
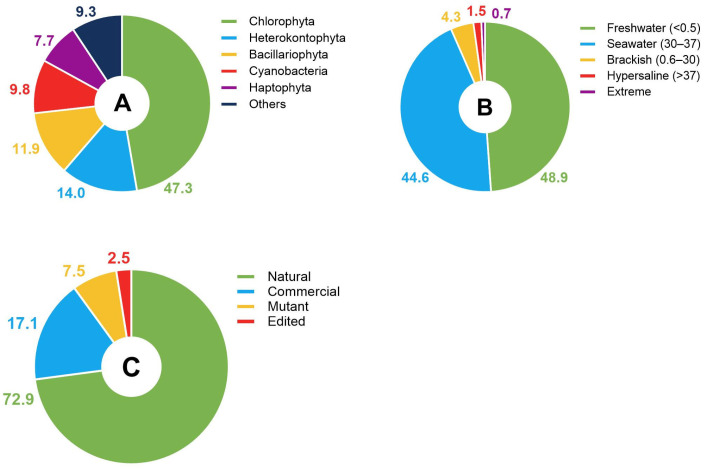
The distribution of microalgae used in publications based on (**A**) group, (**B**) original environment, and (**C**) selection strategy.

Investigating the microorganisms used in recent studies helps to understand which groups of microalgae have been used most commonly and have the greatest potential for applied research in lipid production for industrial sectors (Figure 3A). Green algae (the phylum Chlorophyta) were the most studied group for lipid production (47.3% of the publications). This group is highly diverse and widely known for its biotechnological potential and ability to produce fatty acids [51]. There was also notable representation of brown and golden algae (the phylum Heterokontophyta; 14.0% of the publications) and diatoms (the phylum Bacillariophyta; 11.9% of the publications) regarding their use in biomass and lipid production [52]. Cyanobacteria (9.8% of the publications) include one of the best-known groups of microalgae used in the food industry, Spirulina (*Limnospira* spp. [53]). Groups with less representation showed significant value in more specific contexts, such as Haptophyta, known for the production of wax esters, and Rhodophyta, known for the production of bioactive compounds [54,55]. The general distribution of publications suggests a concentration in more established groups in terms of commercial development, with organisms that are easier to handle and have a higher lipid yield. Five publications covered microalgae as a single group. Among the other records, there were 86 species distributed in 69 genera of microalgae (Supplementary Materials). Among the genera observed, the 10 most represented are *Nannochloropsis* (Heterokontophyta), *Scenedesmus* (Chlorophyta), *Chlorella* (Chlorophyta), *Dunaliella* (Chlorophyta), *Isochrysis* (Haptophyta), *Limnospira* (Cyanobacteria), *Tetraselmis* (Chlorophyta), *Phaeodactylum* (Bacillariophyta), *Chlamydomonas* (Chlorophyta), and *Haematococcus* (Chlorophyta), which together accounted for 61.3% of the publications. The most commonly used species for lipid production with potential in the food industry are *Chlorella vulgaris*, *Phaeodactylum tricornutum*, *Isochrysis galbana*, *Nannochloropsis oceanica*, *Dunaliella salina*, *Nannochloropsis gaditana*, *Haematococcus pluvialis*, *Nannochloropsis oculata*, *Scenedesmus obliquus*, and *Limnospira platensis*.

Around 50% of the organisms used in the retrieved publications are from freshwater, which explains to some extent the contribution of green algae described above. Further, 44.6% of the publications investigated marine microalgae (Figure 3B), including marine diatoms that are known for their high lipid production and significant biotechnological potential [56]. Freshwater and marine environments are more accessible, and organisms found there are easier to cultivate and scale up. On the other hand, the cultivation of organisms from hypersaline or extreme environments (extremophiles) would require more sensitive control, increasing production costs and hindering their viability. As a result, little investment is directed toward these groups, which explains their low contribution to this classification (0.7% of the publications).

The selection of the microalgae was divided into four categories: natural, commercial, mutant and edited (Figure 3C). The majority of the publications (72.9%) focused on natural organisms isolated from their local environment of origin and kept in the laboratory, without active intervention to select the strains. The mutant category includes organisms that have undergone untargeted genetic modification (representing 7.5% of the publications), whereas the edited category included organisms that have undergone genetic editing aimed at a specific objective (2.5% of the publications). For the commercial category, microalgal biomass (dry or wet) was purchased; 17.1% of the publications fall into this category. In these cases, the main focus was on new products and formulations and testing extraction and processing methods. As mentioned above, this finding suggests that the relevance of modern molecular techniques is still limited by their high costs and weak regulatory framework [42].

### 3.3. Cultivation Conditions

Control of abiotic factors is essential to optimize the production of biomass and to target compounds of interest in microalgal cultures. Among the cultivation conditions in the 239 publications, we analyzed the culture media, irradiance, photoperiod, temperature, salinity, and pH. Choosing the right culture medium is fundamental to the successful cultivation of microalgae. It defines the salinity at which the organisms will be grown and provides the nutrients needed. Different culture medium compositions may also act to maximize the production of biomass and compounds of interest. There are currently several recipes for growth media, some even targeted to specific groups. There were 47 culture media reported in the publications, with the presence of both homemade media prepared with available recipes [57], some of commercial origin, and others that are simpler such as sterile water (SW) and artificial seawater (ASW) (Supplementary Materials). The most commonly used media were BG11 [58] in 22.8% of the publications, used for cyanobacteria and other freshwater microalgae; f/2 [59] in 22.2% of the publications, adapted for the cultivation of marine microalgae, often based on collected and sterilized seawater and enriched with the components described in the formulation that are necessary for growth in saline environments; and Bold’s Basal Medium (BBM) [60] in 15% of the publications, for the cultivation of green algae. Changes in nutrient availability, such as nitrogen limitation, supplementation, and changes in the carbon-to-nitrogen ratio, can induce metabolic changes that favor the accumulation of lipids or other compounds of commercial value. This approach is commonly used in biotechnological studies to optimize large-scale production, particularly in the field of bioenergy [61]. Changes in the culture medium were reported in 62 of the 168 publications in which the medium used was mentioned (Table 6).

**Figure 4:**
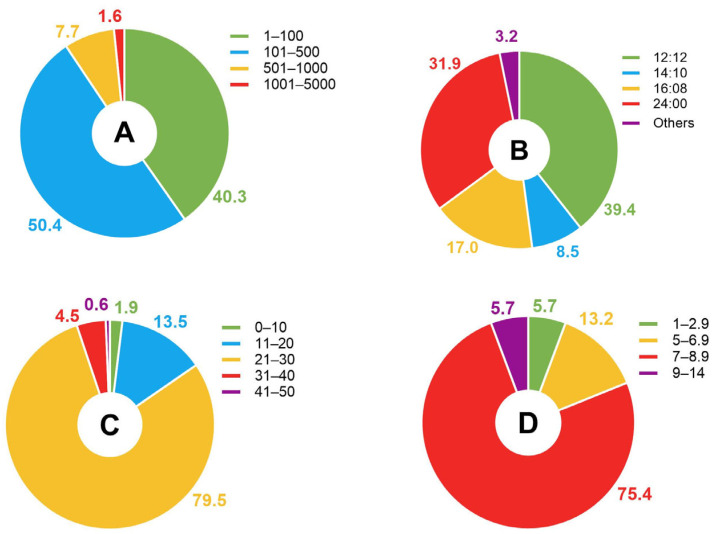
The distribution of cultivation conditions used in the publications based on (**A**) light intensity (µmol photons m^−2^ s^−1^), (**B**) photoperiod, (**C**) temperature (°C), and (**D**) pH.

Photosynthesis is a process that depends on light energy, so its efficiency can be regulated by the amount of light available to the organism. In this sense, both the amount of light (irradiance) and the duration of the illuminated phase of cultivation (photoperiod) are able to regulate the metabolism of microalgae and the synthesis of specific compounds such as lipids [62]. Among the recorded light intensities, the most frequently used intensity was 100–500 µmol photons m^−2^ s^−1^ (60% of the records), followed by 1–100 µmol photons m^−2^ s^−1^ (40% of the records) (Figure 4A). Meanwhile, the publications mentioned continuous (24:00) and intermittent illumination regimes, with the most common regime being 12 h of illumination and 12 h of darkness (12:12; 39.4% of the records). Continuous light regimes were mentioned in 31.9% of the records (Figure 4B).

Temperature affects enzymatic activity and photosynthetic efficiency, and its variation can inhibit or stimulate growth and promote lipid accumulation in response to stress [63]. The publications mentioned a temperature of 6–45 °C. Of the 149 publications mentioning temperature, 79.4% used a temperature of 21–30 °C, and 13.4% used a temperature of 11–20 °C (Figure 4C).

The pH affects the availability and solubility of nutrients in the culture medium, such as nitrates, phosphates, and trace metals, which are essential for cell development [64,65]. In general, most microalgae grow best at a slightly alkaline pH (between 7.5 and 8.5), but different species may have specific requirements. Of the 239 publications analyzed, only 50 mentioned pH when describing cultivation conditions. There were 53 occurrences of pH values, 75.5% of which were between 7 and 8.9 (Figure 4D). In a few cases, an extreme pH such as 2.5 was reported.

### 3.4. Lipids

**Figure 5:**
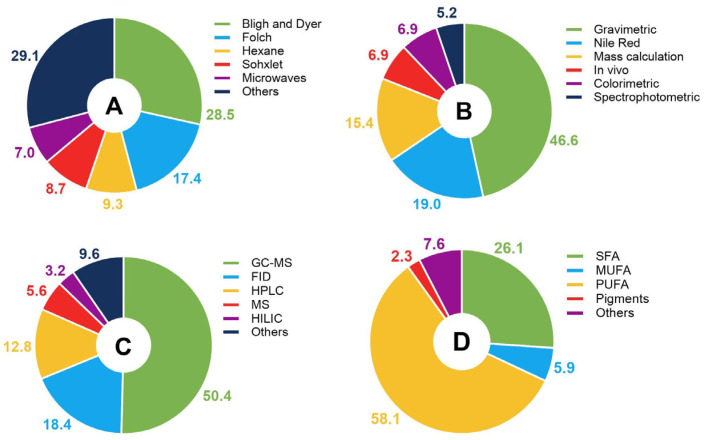
The distribution of lipid processing used in publications based on (**A**) the extraction method, (**B**) the quantification method, (**C**) the characterization method, and (**D**) the lipid class. GC-MS = Gas chromatography-mass spectrometry; FID = Flame ionization detection; HPLC = High performance liquid chromatography; MS = Mass spectrometry; HILIC = Hydrophilic interaction chromatography; SFA = Saturated fatty acids; MUFA = Monounsaturated fatty acids; PUFA = Polyunsaturated fatty acids.

Extraction methods play a critical role in the quality and quantity of lipids obtained from microalgae, directly affecting the efficiency and yield of the process. These methods vary in their ability to disrupt microalgal cell walls and to release intracellular lipids [50]. The choice of extraction method requires consideration of both the type of microalgae and the ultimate goal of the work, depending on the area in which it will be applied, such as nutraceuticals, food, or biofuels. There were 21 different extraction methods mentioned in the 255 publications. The most commonly used techniques for lipid extraction are related to the use of different solvents, such as hexane and alcohol, but a combination of methanol and chloroform stood out, which in many cases is also described as part of the Bligh and Dyer method [66] and the Folch method [67] (Figure 5A). These methods are based on the use of a mixture of polar and apolar solvents to separate lipids from cellular material, but they differ in the proportions of solvents used. Both are widely used because of their high efficiency in extracting large amounts of complex lipids. Another notable extraction technique is the Soxhlet, which is widely used for prolonged lipid extractions and ensures continuous contact between the sample and the extraction solvent. This method accounted for 8.7% of the techniques described in the publications.

The potential of microalgae and the efficiency of extraction methods can be assessed by quantifying the lipids present in the cell or extracted. For this purpose, different techniques are used to quantify the total lipid content of organisms and samples. The publications mentioned six methods, with the greatest contribution from gravimetric quantification (almost 50% of the records), followed by the Nile Red fluorescence method (20% of the records) (Figure 5B). This technique allows the detection of lipids by fluorescence microscopy or flow cytometry.

Characterization of the lipid profile is essential for assessing the potential of microalgae as it determines the composition and quality of the extracted lipids, including the proportion of saturated fatty acids (SFAs), monounsaturated fatty acids (MUFAs), and PUFAs. This analysis is important to identify the ideal lipid profile for different applications where a specific fatty acid composition is desired. In addition, characterization makes it possible to assess the commercial and industrial potential of the crop, helping to optimize cultivation conditions and extraction processes to maximize lipid yields. The analyzed publications mentioned 12 lipid characterization techniques (Figure 5C) from a total of 186 recorded occurrences. The most frequently used technique was gas chromatography coupled to mass spectrometry (GC-MS), which accounted for 50% of the records.

The lipid groups were analyzed at different levels of resolution (Figure 5D)—that is, depending on the technique used in the publication, the characterization was presented as larger groups such as MUFAs and PUFAs, or as the quantification of individual lipid compounds such as α-linoleic acid and palmitic acid. SFAs accounted for 26.1% of all lipid component records. This group is important to the food industry because of its thermal and oxidative stability, which ensure a longer shelf life for processed foods. Omega-3 and omega-6 fatty acids are considered essential for human health because the human body cannot synthesize them naturally [33,68]. These lipids, as well as omega-9 fatty acids have attracted industrial interest because of the health benefits associated with their consumption, particularly in the prevention of cardiovascular disease and in the fight against inflammation. In particular, omega-3 and omega-6 fatty acids are often incorporated into functional products, dietary supplements, and fortified foods [33,68]. A more detailed analysis of the components of major lipid groups revealed the following mentions in the publications: 13 SFAs; 9 MUFAs, of which 3 belong to the omega-9 class; 16 PUFAs, of which 5 belong to the omega-3 class and 5 belong to the omega-6 class; five pigment compounds; and another 17 compounds grouped together to form the group of other lipids (Supplementary Materials). Although numerous studies have been found related to microalgae lipids and their potential applications in the food sector, no evidence was found that saturated microalgae oils are used as a substitute for saturated animal fat in feed. Evidence of the promising replacement of animal oil with microalgae oil in *Sparus aurata* diets was found [69], but no evidence was found outside the aquaculture area.

## 4. Conclusions

The results highlighted the importance of microalgae as a promising biotechnological solution for the production of lipids in the food industry, despite a decrease in publications in 2023. Most publications were from China and reported lab-scale, non-applied research focused on agricultural and life sciences. Freshwater chlorophytes (green algae) with no gene editing were the most used organisms for lipid production in the publications. This group is widely used in various industrial sectors, which highlights its biotechnological potential. Despite being relatively easy to cultivate, several studies focused on improving and optimizing microalgal cultivation conditions to accumulate specific high-value compounds. Whereas one of the main tools used to stimulate lipid production is the limitation of nutrients (mainly nitrogen) in culture media, several studies explored the differences in temperature (21–30 °C), irradiance (101–500 µmol photons m^−2^ s^−1^), photoperiod (12:12), and pH (7–8.9).

Lipids were mainly extracted using the Bligh and Dyer or Folch method, quantified gravimetrically, and characterized using GC-MS. Publications referring to PUFAs, such as omega-3 and omega-6, were the most abundant. These results were expected because these lipids are already recognized for their health benefits. On the other hand, SFAs were also significantly represented in the analyzed publication. The dominant presence of these two groups confirms their potential in the production of food ingredients derived from microalgae, with an emphasis on their applicability in plant-based products. The scientific evidence found in the mapping shows that research on microalgae fatty acids is mostly focused on laboratory scales. Evidence of the use of microalgae oils in food is concentrated in aquaculture. Therefore, we can observe a window of opportunity for deeper research into the topic of saturated fatty acids in food, especially in plant-based foods, as the field of scientific knowledge in this area is wide open, and their potential uses are presented here in this scientific mapping.

## Figures and Tables

**Table 1:** Keyword combinations used in the search and the main focus of their results.

Keywords Combination	Focus
Microalgae + Oil	Product
Microalgae + Lipids	
Microalgae + Fatty + Acid	
Microalgae + Saturated + Oil	
Microalgae + Oil + Food	Applications in the food sector
Microalgae + Lipids + Food	
Microalgae + Fatty + Acid + Food	
Microalgae + Saturated + Oil + Food	
Microalgae + Oil + Food + Perspective	Future trends for microalgae oil applications in the food sector
Microalgae + Lipids + Food + Perspective	
Microalgae + Fatty + Acid + Food + Perspective	
Microalgae + Saturated + Oil + Food + Perspective	
Microalgae + Oil + Food + Trends	
Microalgae + Lipids + Food + Trends	
Microalgae + Fatty + Acid + Food + Trends	
Microalgae + Saturated + Oil + Food + Trends	
Microalgae + Oil + Trends	Future trends for microalgae oil applications in general
Microalgae + Lipids + Trends	
Microalgae + Fatty + Acid + Trends	
Microalgae + Saturated + Oil + Trends	

**Table 2:** Total number and percentage of scientific publications per country.

Country	No. of Publications	%
China	42	13.1
Spain	18	5.6
Brazil	17	5.3
Italy	17	5.3
Portugal	17	5.3
India	16	5.0
USA	13	4.1
Japan	12	3.8
France	11	3.4
Iran	10	3.1
Malaysia	10	3.1
South Korea	10	3.1

Country assignment was based on the first author.

**Table 3:** Total number and percentage of scientific publications per subject.

Subject Area	No. of Publications	%
Agricultural and life sciences	114	24.8
Biochemistry and genetics	62	13.5
Chemical engineering	53	11.5
Environmental sciences	40	8.7
Chemistry	37	8.1
Microbiology and immunology	36	7.8
Energy	32	7.0
Engineering	16	3.5
Pharmacology, toxicology, and pharmaceuticals	16	3.5
Medicine	11	2.4
Multidisciplinary studies	8	1.7
Social sciences	8	1.7
Health	6	1.3
Computational sciences	5	1.1
Planetary and earth sciences	4	0.9
Material sciences	4	0.9
Nursing	2	0.4
Veterinary sciences	2	0.4
Economics	1	0.2
Mathematics	1	0.2
Physics and astronomy	1	0.2

**Table 4:** Total number and percentage of scientific publications per application.

Application	No. of Publications	%
Basic research	121	27.0
Food	93	20.7
Bioenergy	64	14.3
Nutraceuticals	60	13.4
Animal feed	37	8.2
Biorefinery	29	6.5
Pharmaceuticals	23	5.1
Aquaculture	16	3.6
Cosmetics	6	1.3

**Table 5:** Total number and percentage of scientific publications per objective.

Objectives	No. of Publications	%
Production and product improvement	157	20.1
Lipid profile characterization	156	20.0
Cultivation conditions	118	15.1
Biomass production	72	9.2
Bioactive compounds	65	8.3
Extraction methods	56	7.2
Benefits from microalgae	53	6.8
Molecular mechanisms	42	5.4
Downstream processes	28	3.6
Lipid detection	28	3.6
Others	7	0.9

**Table 6:** The total number and percentage of scientific publications per growth medium treatment.

Growth Medium Treatment	No. of Publications	%
Nutrient supplementation	27	43.5
Nutrient starvation	27	43.5
Nutrient limitation	8	12.9

## Data Availability

The original contributions presented in this study are included in the article/Supplementary Materials. Further inquiries can be directed to the corresponding author.

## Supplementary Materials

The following supporting information can be downloaded at: https://www.mdpi.com/article/10.3390/foods14193451/s1.

## Abbreviations

The following abbreviations are used in this manuscript:

DHA	Docosahexaenoic acid
EPA	Eicosapentaenoic acid
FID	Flame ionization detection
GC-MS	Gas chromatography-mass spectrometry
HILIC	Hydrophilic interaction chromatography
HPLC	High performance liquid chromatography
MUFA	Monounsaturated fatty acid
PUFA	Polyunsaturated fatty acid
SFA	Saturated fatty acid
TAG	Triacylglycerol
